# Supplementary material for: Splice-Junction-Based Mapping of Alternative Isoforms in the Human Proteome
Source: Cell Rep. Author manuscript; Available in PMC 2020 Jan 15. (PMC6961840; doi:10.1016/j.celrep.2019.11.026)
Supplement: 2 [file NIHMS1546469-supplement-2.zip › DF1/PXD010154/Heart/Heart_87_TPM3_AISEELDHALNDMTSG.pdf]

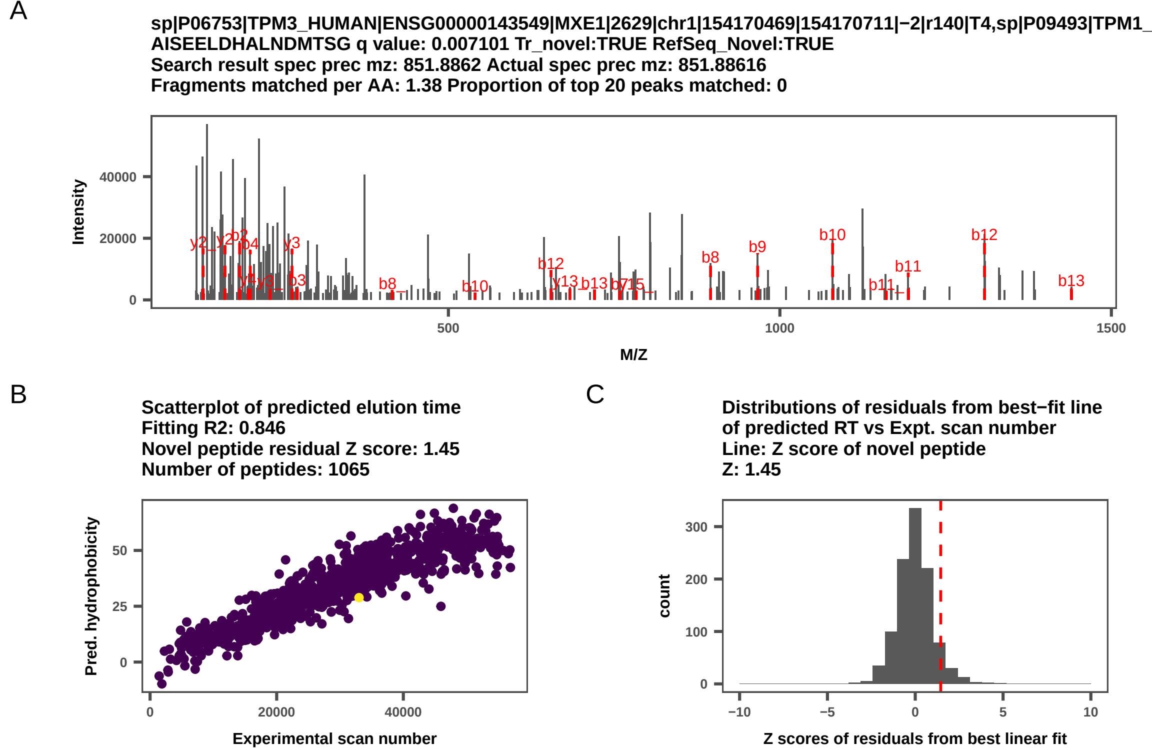
A
sp|P06753|TPM3_HUMAN|ENSG00000143549|MXE1|2629|chr1|154170469|154170711|−2|r140|T4,sp|P09493|TPM1_
AISEELDHALNDMTSG q value: 0.007101 Tr_novel:TRUE RefSeq_Novel:TRUE
Search result spec prec mz: 851.8862 Actual spec prec mz: 851.88616
Fragments matched per AA: 1.38 Proportion of top 20 peaks matched: 0
Intensity
0
20000
40000
M/Z
500
1000
1500
b8
b9
b10
b11
b12
b13
B
Scatterplot of predicted elution time
Fitting R2: 0.846
Novel peptide residual Z score: 1.45
Number of peptides: 1065
Pred. hydrophobicity
0
25
50
Experimental scan number
0
20000
40000
C
Distributions of residuals from best−fit line
of predicted RT vs Expt. scan number
Line: Z score of novel peptide
Z: 1.45
count
0
100
200
300
Z scores of residuals from best linear fit
−10
−5
0
5
10
